# The impacts of synthetic and cellulose-based fibres and their associated dyes on fish hosts and parasite health

**DOI:** 10.1007/s11356-023-30794-0

**Published:** 2023-11-13

**Authors:** Scott MacAulay, Numair Masud, Josh Davies-Jones, Benjamin D. Ward, Jo Cable

**Affiliations:** 1 School of Biosciences, Cardiff University, Cardiff, CF10 3AX UK; 2 School of Chemistry, Cardiff University, Cardiff, CF10 3AT UK

**Keywords:** Fibre pollution, Microplastic, Freshwater fish health, Parasite resistance

## Abstract

Plastic pollution is now a ubiquitous feature of freshwater systems and the majority of this is fibrous. Here, we test the effects of plastic and cellulose-based fibres (polyester, cotton, and bamboo from commercial clothing) on fish host-parasite interactions using a freshwater fish host-parasite model system (guppy *Poecilia reticulata*-*Gyrodactylus turnbulli*). For uninfected fish, polyester exposure was associated with significantly higher mortality rates compared with the other two fibre types. For infected fish, whilst polyester and cotton exposure were not associated with any significant changes to parasite burdens, fish exposed to bamboo fibres had significantly reduced maximum parasite burdens compared with fish not exposed to any fibres, indicating that the bamboo fibres and/or associated dyes conferred some degree of resistance or tolerance. Whilst unable to determine the exact nature of the chemical dyes, when testing off-host parasite survival on exposure to the fibre dyes, cotton and particularly polyester dyes were associated with higher parasite mortality compared to bamboo. Overall, we add to the growing body of evidence which shows that polyester microplastic fibres and their associated dyes can be detrimental for both fish and parasite survival, and we highlight the need for increased transparency from textile industries on the chemical identity of fabric dyes.

**Supplementary Information:**

The online version contains supplementary material available at 10.1007/s11356-023-30794-0.

## Introduction

Textile industries are key players contributing to the ever-increasing burden of particulate pollution, especially microplastics, an ecological issue that has recently taken centre stage. Over 80% of environmental microplastics (plastics < 5 mm) are fibrous (freshwater: Horton et al. 2017; marine: Vince and Stoett 2018; terrestrial: Rillig and Lehmann 2020) and 35% of oceanic microplastic pollution is attributed to the fashion industry that produce garments from non-degradable synthetic polymers (United Nations Climate Change 2018). Fast fashion produces cheap, low-quality clothing per year (Young 2021) providing affordable fashion to those on a budget, but such garments are only worn on average ten times before being discarded and deposited in landfills (Barnardos 2015; TRAID 2018). Fibres from discarded garments typically persist in the environment, many ending up in water bodies through run-off, and are incorporated throughout the food web (De Falco et al. 2019). The sheer scale of fibre waste generation is concerning, with a single-household wash cycle releasing 100–300 mg of fibres per kg of fabric (De Falco et al. 2019). Scaling this up leads to exorbitant quantities; estimates in Finland, for example, reveal annual household production of 154,000 kg for synthetic polyester fibres and 411,000 kg for cotton fibres (Sillanpää and Sainio 2017). Whereas natural polymers are degraded by microbes (Pekhtasheva et al. 2012; Arshad et al. 2014), synthetic fibres are resistant to such breakdown (Resnick 2019) so greater use of natural polymers is one mitigation strategy in the global initiative to reduce non-degradable plastic pollution. It is essential, however, to assess whether these products marketed as ‘ecologically friendly’ are less harmful for organisms when released into multiple habitats. Indeed, one of the key issues raised in response to the EU Directive 2019/904 is giving a green light to non-plastic polymers, such as bamboo, hemp, or fruit fibres, before their biological impact is assessed (European Environment Agency 2022).

To date, biological assessments of fibre exposure and/or consumption are mostly limited to invertebrates and reveal no clear trend and typically focus on plastic fibres, ignoring cellulose-based fibres such as cotton, rayon, and bamboo (Suran 2018). Consumption of polyester fibres by earthworms (*Lumbricus terrestris*) revealed no effect on mortality or avoidance behaviour (Prendergast-Miller et al. 2019), whereas land snails (*Achatina fulica*) exposed to polyethylene terephthalate (PET) fibres showed reduced feeding ability and increased oxidative stress (Song et al. 2019). For vertebrates, fibre pollution has been detected in the guts of many animal groups including fish (Lusher et al. 2015), birds (Lourenço et al. 2017), and mammals (Lusher et al. 2015), but only two studies to the best of our knowledge have assessed the direct biological effects of fibres on fish health. Adult Japanese medaka (*Oryzias latipes*) exposed to polyester and polypropylene fibres for 21 days were not impacted in terms of reproductive changes and mortality, but did show aneurysm in lamellae, opercular swelling, and abnormal mucosal cell proliferation (Hu et al. 2020). Juvenile Chinook salmon (*Oncorhynchus tshawytscha*) that were exposed shorter term (10 days) to polyester fibres showed no changes in gut mass (Spanjer et al. 2020). Fibre pollution could also impact fish health by reducing resistance to parasitic infections; however, this has yet to be tested. Indeed, it is well known that pollutants can directly impact fish resistance to parasitic disease by influencing underlying immunity (reviewed in Tort 2011). Microplastics, which are a key component of fibre pollution within aquatic environments, can impact fish immunity by impacting the regulation of gene expression and immune cells (Limonta et al. 2019; Zwollo et al. 2021), and have been shown to reduce resistance to parasitic infections in freshwater sticklebacks (*Gasterosteus aculeatus*; see Masud et al. 2022).

Here, we investigate the impact of plastic and cellulose-based fibres (polyester, cotton, and bamboo) on fish host-parasite interactions. Synthetic polyester and cotton were chosen as they are two of the most used fibre types for clothing material (Carr 2017). The durability of synthetic polyesters makes them resistant to natural degradation (Carr 2017), whereas cotton is a natural fibre crop, but with a large water footprint (Chapagain et al. 2006). Bamboo, on the other hand, requires substantially less water than cotton and is also a natural polymer. Therefore, a key question here is whether cellulose-based fibres are ‘better’ in relation to their potential impact on fish welfare compared with polyester fibres. Furthermore, we assessed whether dyes associated with these fibres had any impacts on the independent (i.e. off host) survival of parasites. We hypothesised that polyester fibres would be associated with increased disease susceptibility, whereas the cellulose-based fibres (cotton and bamboo) would have more attenuated impacts on fish welfare, and dyes associated with the tested fibres, would impact off-host parasite survival. To test these hypotheses, we used an established host-parasite system, the guppy (*Poecilia reticulata*)-*Gyrodactylus turnbulli* model*.*

## Materials and methods

### Host-parasite system

**Fig. 1:**
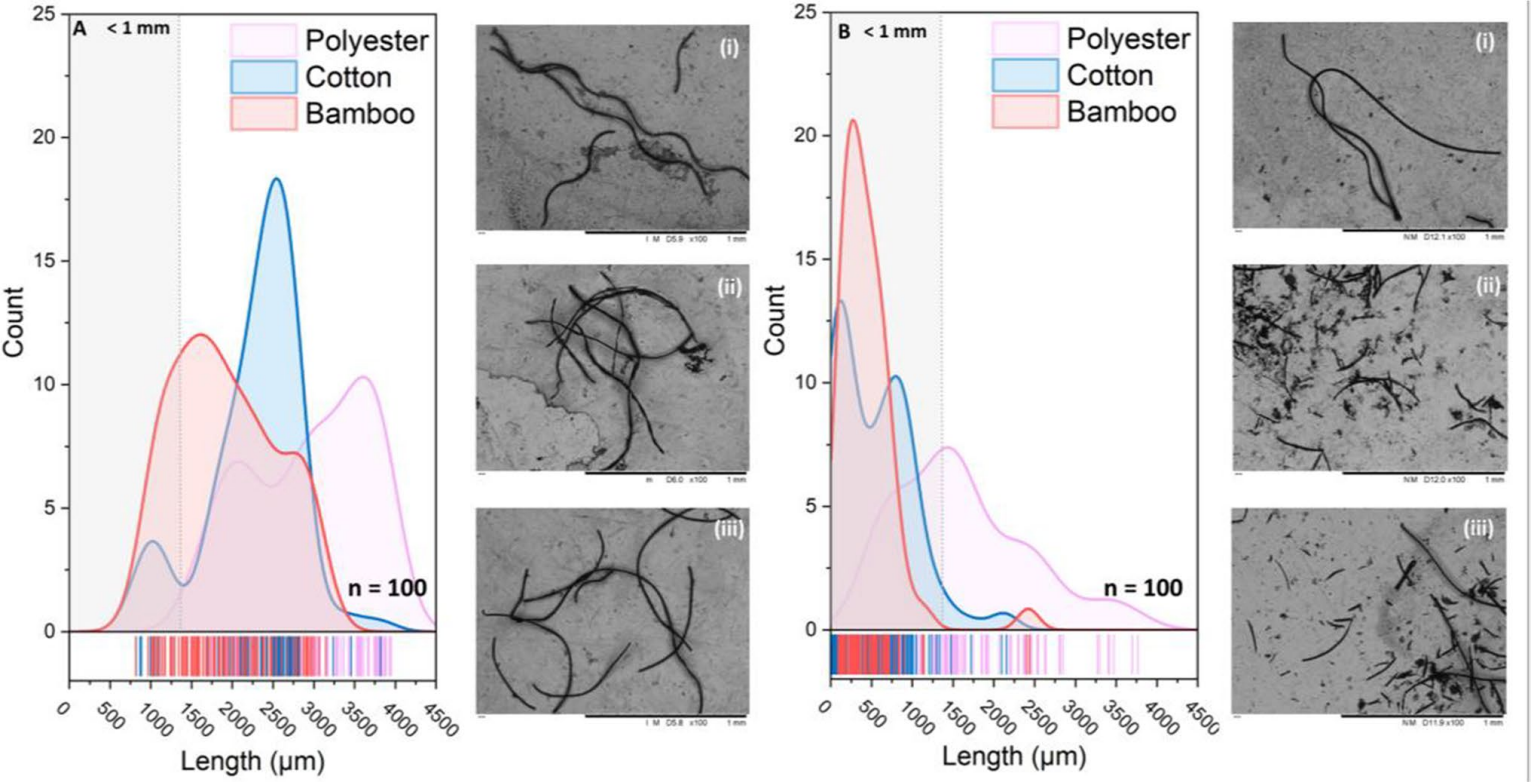
Particle size distribution and Scanning Electron Micrographs of (i) polyester, (ii) cotton, and (iii) bamboo fibres shed after 24 h (**A**) and 60 days in water (**B**). In A, the mean particle sizes with standard deviation of the fibres are 2933.7 μm ± SD 723.5 for polyester, 2273.7 μm ± SD 566.8 for cotton, and 1867.7 μm ± SD 605.4 for bamboo. In B, the equivalent values are 1615.4 μm ± SD 803.4 for polyester, 539.1 μm ± SD 463.6 for cotton, and 446.8 μm ± SD 364.6 for bamboo. Note the physical degradation of fibres between panels A and B is more apparent for cotton and bamboo compared to polyester

The guppy is a tropical fish species, native to the Caribbean Islands and Venezuela, and an invasive non-native species on every continent except Antarctica (Magurran 2005). The genus, *Gyrodactylus*, is a species rich group of fish parasites that are ecologically and economically important (Bakke et al. 2007). The primary monogenean ectoparasite *G. turnbulli* is a major pest in the ornamental fish trade (Cable 2011). For this investigation, we used size-matched, mature, mixed ornamental male guppies (*Poecilia reticulata* from Guppy Farm UK—6–8 months old). Upon arrival at Cardiff University, all fish (*n* = 240) were acclimated for 24 h in groups (~ 10–15 individual fish) within 40-L aquaria at 24 ± 0.5 °C on a 12:12 light/dark photoperiod. All fish were then confirmed as ectoparasite free, via three daily consecutive microscopic screens (see Schelkle et al. 2009). Briefly, this involved mildly anaesthetising individual fish using 0.02% MS-222 and observing the surface of each fish for visible signs of parasitaemia (e.g. raised fins, white spots, abnormal growths) and any infected fish were excluded. To rule out bacterial infection, all fish were treated with the antibacterial treatment (Myxazin) as per manufacturer instructions (Waterlife) 2 weeks prior to the start of the experiment. We acknowledge that chemical derivatives from the Myxazin treatment may have persisted in the tissue of the experimental fish, but as our model parasite is an epidermal feeder, any tissue-based toxicity is unlikely to have impacted the parasites. Furthermore, all fish (controls and fibre treatments) were exposed to Myxazin. For experimental infections, we used the *Gt3* strain of *Gyrodactylus turnbulli*, isolated from a Nottingham aquarium pet store (King and Cable 2007; see Fig. 1A). This parasite has been cultured under laboratory conditions since establishment in November 1997. All fish, prior to experimental infections, were weighed on an electronic scale by mildly anaesthetising individuals with 0.02% MS-222, and fish were then weighed again at day 52 (i.e. end of experiment) to determine if fibre exposure and consumption impacted wet mass.

### Fibre preparation, exposure, and chemical analysis

Fish were separated into four treatment groups: (1) control (*n* = 60), (2) polyester fibre (*n* = 60), (3) cotton fibre (*n* = 60), and (4) bamboo fibre (*n* = 60). For the polyester and cotton fibres, we sourced 100% polyester and 100% cotton shirts, respectively. The bamboo fabric consisted of 95% bamboo viscose and 5% elastane. Due to the structural property of bamboo (i.e. short inflexible fibres), 100% bamboo is typically not utilised for commercial bamboo clothing and elastane is added to ensure flexibility of finalised fabrics (Muthu 2017). All materials were black (to avoid colour as a confounding variable) and obtained from commercial retailers. Whilst we were unable to ascertain the exact nature of the dye (due to commercial sensitivity), the most commonly used black dye is Reactive black 5 (industrial name Setazol Black) and therefore the most likely to be found in the fabrics tested in this experiment (Bilińska et al. 2016; Al-Tohamy et al. 2020).

The fabrics were first cut into 7.5-cm^2^ squares, then shred into 1.5-cm^2^ pieces using sterile scissors and immersed in 1 L of aquarium water (i.e. the same water used for fish tanks mentioned above), and agitated to promote fibre shedding to simulate a washing cycle. After 24 h, the fibre water was aliquoted into 50-mL bottles and this stock solution was used throughout the study for dosing fish; therefore, by the end of the experiment the fibres had been soaked for 52 days. A drop of fibre water (50 μL) was then viewed under a compound microscope and the number of fibres counted. This was repeated 10 times per fibre treatment (fibre number ranged for bamboo 22–52, cotton 22–50, and polyester 21–65) to calculate the mean number per 1 mL, which was approximately 700 fibres/L. The size distribution of fibres used in this study was determined using a Titachi TM3030 Plus benchtop microscope in Back Scattering Electron (BSE) mode at 15 kV. To isolate the fibres from the solution, a centrifuge was used to spin 50 mL of soaked fibre solution at 4000 × *g*. The resulting pellets were washed with deionised water three times and resuspended in 5 mL. To provide optimal contrast for fibre identification, three drops of this highly concentrated solution from each treatment were sequentially drop cast onto a steel disc. We counted a total of 100 fibres from each treatment and plotted a particle size distribution with a Gaussian kernel-smooth curve fit (see Fig. 1).

Our analysis showed that the mean fibre length of all three fibre types decreased with prolonged soaking time, with a greater decrease observed in cotton and bamboo fibres than polyester fibres. After a 24-h soak, the average fibre length was 2933.7 μm for polyester, 2273.7 μm for cotton, and 1867.7 μm for bamboo. This decreased further by the end of the experiment and measured at 60 days to be 1615.4 μm for polyester, 539.1 μm for cotton, and 446.8 μm for bamboo (Fig. 1). Additionally, we observed evidence of particle degradation in the micrographs of cotton and bamboo fibres. In contrast, although some bowing, and roughness could be seen on the surface of polyester fibres, there were no clear areas of degradation evident. For this study we were unable to quantify the rate of fibre degradation. As guppies are gape limited predators (Magurran 2005) and we know that the fibres degrade and decrease in size over time, the male guppies in this study with a gape of diameter range 1.5–2.5 mm would have been able to consume the fibres.

**Fig. 2:**
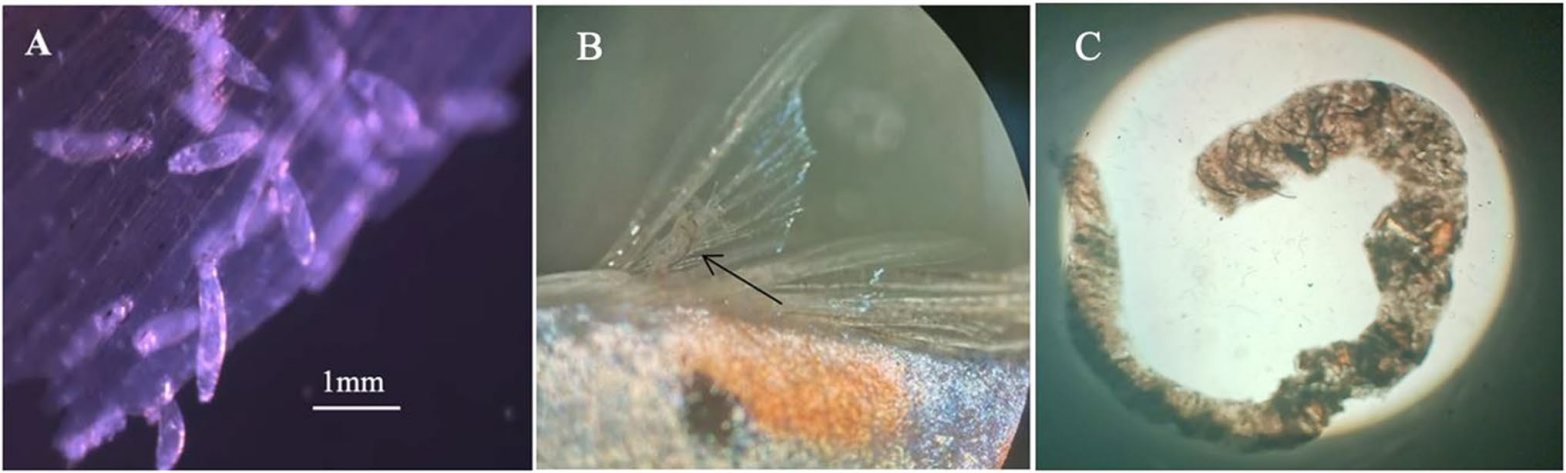
The host-parasite system with fibre optic illumination. **A** *Gyrodactylus turnbulli* individuals on the caudal fin of guppy (*Poecilia reticulata*) under × 10 magnification. **B** Fibre (arrow) being egested by male guppy at × 2 magnification. **C** Faecal pellet of a guppy with a fibre encased within at × 4 magnification, indicating the passage of fibres through the guppy gastrointestinal tract. A 1-mm scale bar shown for all pictures

A preliminary trial was conducted on *n* = 5 fish per fibre treatment where individual fish were maintained in 1-L containers. Fish were exposed for 7 days to ~ 700 fibres/L, equivalent to fibre loads found in some natural environments (Carr 2017). This involved adding 1 mL of the fibre mixture at the same time as adding flake food (Aquarian®) to each 1-L container. Control fish (*n* = 5) were maintained under the same conditions but without fibre exposure. Each day, faecal matter from the water was transferred using a glass pipette into a pre-cleaned glass petri dish and then dissected under a dissecting microscope to count the number of fibres encapsulated within the faeces. Fibres were clearly observed within all faeces of fish exposed to fibres even within the first 24 h of exposure (fibre range = 12–23 per faecal pellet; see Fig. 2B and C).

For the main experiment, all fish were isolated into 1-L containers (i.e. 1 fish per 1-L container) and fish were exposed to fibres (i.e. ~ 700 fibres/L) for 21 days. This time frame of exposure was chosen as it corresponds to significant effects seen in changes to host-parasite responses to granular microplastic exposure on the guppy-*G. turnbulli* system (Masud and Cable 2023). Control fish were fed the same quantity of flake food (2% of body weight) without fibre addition, to ensure that nutrition was not a confounding variable. Due to this immersion mode of fibre exposure, it is likely that consumption of fibres occurred passively. A full water change (for the preliminary trial and the main experiment fish) occurred every alternate day coinciding with feeding, which involved removing all water from the 1-L containers in which fish were housed and replacing with fresh temperature-controlled dechlorinated water. It is acknowledged that water changes may cause some degree of stress to fish; however, as this cleaning method was applied across all treatments using the same technique, any potential confounding effects of handling stress would average out. During feeding, all precaution was taken to ensure that the experimenters’ clothing did not contribute to fibre contamination by ensuring short-sleeved clothing was worn during all feeding regimes. However, we acknowledge that even under tightly controlled laboratory conditions atmospheric fibre contamination is possible (Gwinnett and Miller 2021), but would have been consistent across treatments.

## Experimental infection

After 21 days of fibre exposure, half of the fish in each treatment group were infected (*n* = 30) and half remained uninfected (*n* = 30). Fish to be infected with *G. turnbulli* were lightly anaesthetised with 0.02% MS-222 and then held in water alongside a donor fish. Using a dissecting microscope with fibre optic lighting, transfer of two gyrodactylid worms to the caudal fin of the recipient fish was observed following the standard methods of King and Cable (2007). Uninfected fish were anaesthetised and handled in the same manner without the introduction of parasites to control for any handling stress (sham infections). All infected and sham infected fish were maintained within 1-L containers throughout the experiment to ensure that transmission was not a confounding variable for this experiment. Parasite numbers were assessed every 48 h for 31 days and this involved mildly anaesthetising infected fish (using 0.02% MS-222) and counting the number of worms present under a dissecting microscope with fibre optic illumination (see King and Cable 2007 for detailed description). Infection monitoring terminated at 31 days as this was the time point at which all fish had either cleared their infections and/or mortality of hosts occurred. As we were able to follow infection trajectories on the same fish over time, pseudo-replication was taken into consideration when statistically analysing parasite data from each fish/replicate (see “Statistical analysis” section below). Fish were categorised as either resistant (parasite numbers on a host fail to increase and individual hosts often managed to clear their infections), susceptible (parasite numbers consistently increase), or responder (parasite numbers increase but then plateau or decrease) (see Bakke et al. 2002 for more in-depth explanation of these categories). The same feeding regimes continued during the infection phase of the experiment, i.e. both fibres and flakes. Any host mortalities were recorded throughout the study.

### Gyrodactylus turnbulli off-host survival

As all fibres within our stock solution released dyes into the water (see Supplementary Material for chromatographic analysis of dyes), we wanted to determine the effect of any chemical dye exposure on off host parasite survival. To investigate this, 40 wells of sterile 96-well plates were inoculated with 100 µL of the same liquid that the fish were exposed to during each fibre treatment (i.e. stock solution) as these contained any leached-out dyes. Individuals of *G. turnbulli* were removed from a recently sacrificed infected fish by gentle agitation with a needle and transferred to the prepared wells via pipette. Worms exposed to fibre dye treatments (*n* = 120, 40 worms per treatment) and control worms exposed to dechlorinated water (*n* = 40) were observed and survival monitored every hour under a dissecting microscope. Any worms that died (mortality confirmed via worm immobility and non-responsive to pin touch) within the first hour were excluded from the experiment. Survival was then monitored until the last *G. turnbulli* worm died at 33 h; hour of death was noted for every expired worm.

### Statistical analysis

All statistical analyses were conducted in R Studio Version 1.3.1073. When analysing host infections, the following response variables were considered: maximum parasite burden, peak parasite day, Area Under Curve (AUC), rate of parasite increase, and host disease status. Here, maximum parasite burden is defined as the maximum number of *G. turnbulli* worms at a particular time point (day), defined as peak parasite day. To calculate AUC, a common parasite metric quantifying total parasite burdens over the course of an entire infection trajectory, we used the trapezoid rule (White 2011). Rates of parasite increase, indicative of parasite reproduction, were calculated as the slope of the curve of individual infection trajectories.

To analyse host infection responses, we used Generalised Linear Models and Generalised Linear Mixed Models (GLMs and GLMMs). For analysing maximum parasite burdens, we used a GLM with a negative binomial error family and a square root link function, within the *MASS* package in R Studio. When analysing peak parasite day and AUC, a GLM with a Gamma error family and log link was used. For rates of parasite increase we used a GLMM within the *lme4* R package to prevent pseudo-replication as rates of parasite increase were calculated on each fish at multiple time points. For the GLMM we used a Gamma error family and a square root link function. When analysing host disease categories (i.e. fish that were categorised as either resistant, susceptible, or responders; see methodology above for more details), a GLM with a Poisson error family and log link function was used. A GLM with a Gaussian error family and log link function was used to determine if there was a significant association between fish wet mass change before and after fibre exposure and the type of fibre used, where the difference in mass was the response variable and fibre treatment was the independent variable. After all model assumptions were met (i.e. normality of residuals and homogeneity of variance), all final models were chosen based on the lowest AIC values (Thomas et al. 2013).

Kaplan–Meier survival analyses were also conducted for in vitro effects of fibres and/or chemical dye exposure on parasite and host survival (infected and uninfected groups). For analysing parasite survival, we used a parametric model and a Cox-proportional hazard model for host survival, and in this regard, we used the R *survival* package. To visualise survival data, we plotted a probability distribution using the *survminer* and *ggfortify* work package. All final survival models were chosen based on the lowest AIC values.

## Results

### Host and parasite survival

**Fig. 3:**
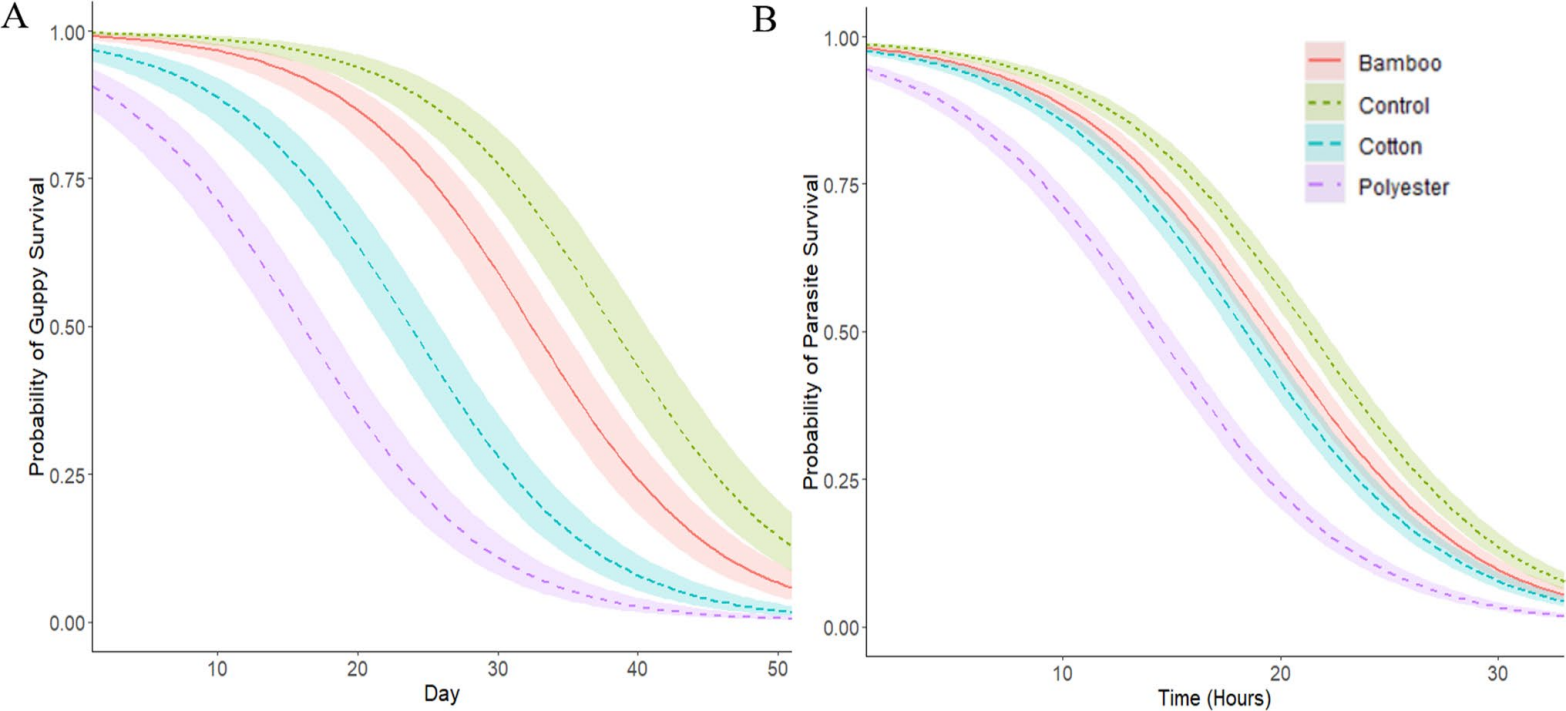
Host (guppy, *Poecilia reticulata*) and off-host parasite (*Gyrodactylus turnbulli*) survival. **A** Prediction curve shows the probability of uninfected *P. reticulata* survival over 52 days of fibre exposure. All mortalities were monitored daily for 52 days with *P. reticulata* isolated in individual 1-L pots. **B** Prediction curve shows the probability of *G. turnbulli* in vitro survival over 33 h of fibre exposure in 96-well microtiter plates

For infected fish, there was no significant difference in mortality between any of the infected treatments related to fibre exposure and/or consumption (*p* > 0.05; see Supplementary Material for detailed statistical outputs). When analysing mortality of uninfected fish, those exposed to polyester suffered significantly earlier mortality compared with control uninfected fish (coef = 2.06, exp(coef) = 7.85, *p* = 0.004; see Fig. 3A for prediction plot), whilst there was no significant difference for cotton or bamboo exposed fish. No significant association was found between the change of fish body mass over 52 days of fibre exposure and the type of fibre that the fish were exposed to (*p* > 0.05). No mortalities were observed in control uninfected fish (i.e. not exposed to any fibres/dyes or parasites).

Gyrodactylid parasites removed from their hosts and exposed to polyester and cotton fibre dyes in vitro died significantly earlier compared to control parasites in just dechlorinated water (i.e. with no dye addition, cotton: *z* =  − 2.58, SE = 0.11, *p* = 0.01; polyester: *z* =  − 3.35, SE = 1.55, *p* = 0.0008). In contrast, exposure to bamboo dye solutions in vitro did not impact parasite survival (Fig. 3B).

### Host disease response

**Fig. 4:**
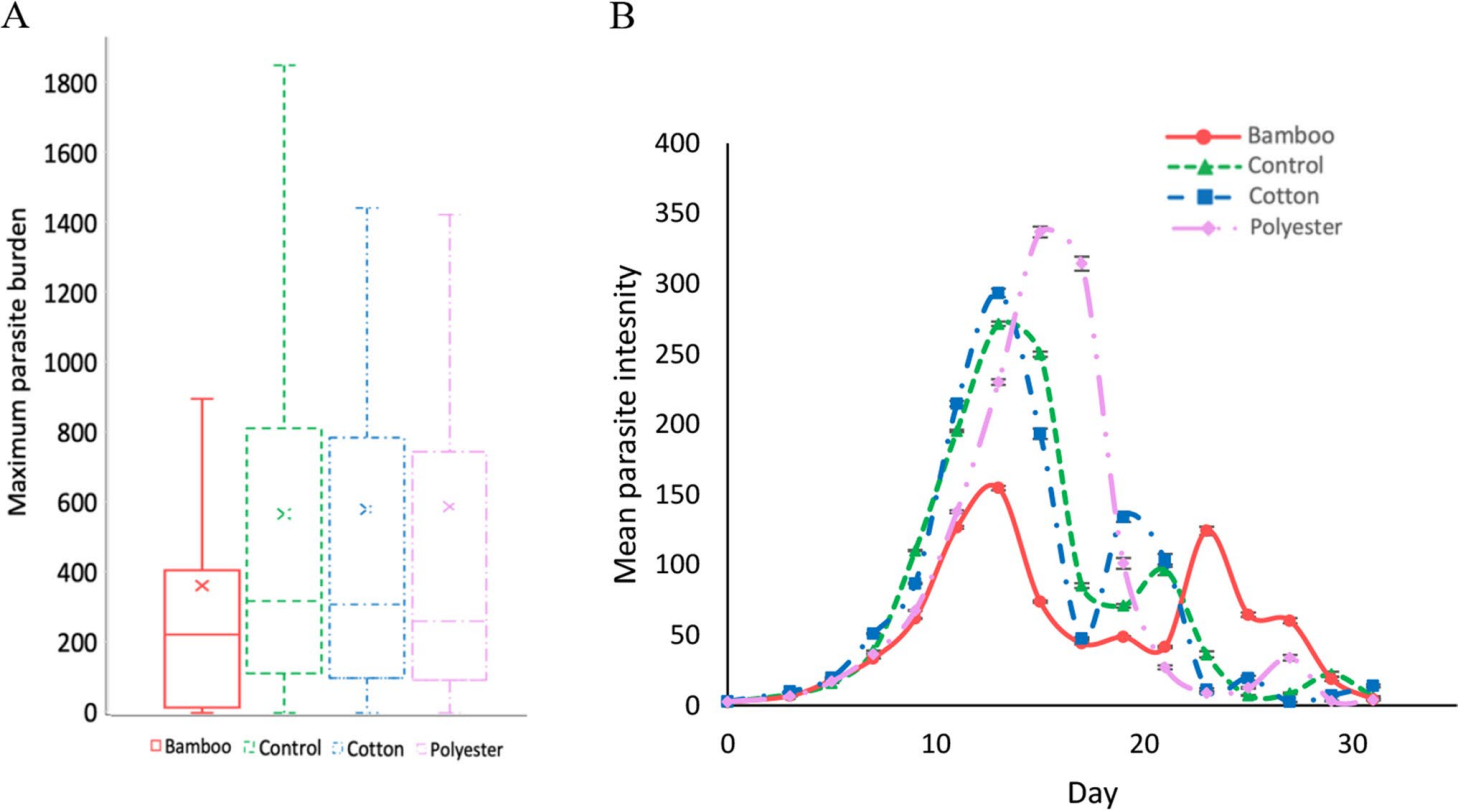
Infection dynamics of guppies exposed to fibres and subsequently infected with *Gyrodactylus turnbulli*. **A** Box plot distribution with mean marker (cross), median line, and inter-quartile range of maximum parasite burdens of guppies (*Poecilia reticulata*) exposed to fibres for 21 days and then infected with *G. turnbulli*. **B** The mean parasite intensity with standard error over the entire 31-day infection trajectory for each fibre exposure treatment. The shape of the smoothed function for each treatment is influenced by mortality of hosts and clearance of parasites

Fish exposed to bamboo fibres had the lowest maximum parasite burdens compared with control fish not exposed to any fibre (GLM: Est. =  − 3.15, SE = 0.97, *p* = 0.001; Fig. 4A). All other fish within the remaining fibre treatments (cotton and polyester) did not significantly vary in their maximum parasite burdens when compared with control fish (see Table 1 for all test statistic outputs). Polyester-exposed fish achieved the highest mean parasite intensity compared with bamboo exposed which achieved the lowest (Fig. 4B), but these were not statistically significant. Peak parasite burden occurred significantly later in bamboo and polyester-exposed fish compared with control infected fish (GLM: bamboo, Est = 0.17, SE = 0.03, *p* < 0.001; polyester, Est = 0.08, SE = 0.03, *p* = 0.01). Supporting this, rates of parasite increase were significantly lower for fish in the bamboo treatment compared with the controls (GLMM: bamboo, Est. =  − 1.95, SE = 0.85, *p* = 0.02). When analysing total parasite burdens over time using AUC metrics though, there was no significant difference between any of the treatments (*p* > 0.05; see Fig. 4B and Table 1). Furthermore, in terms of host disease categories (i.e. resistant, susceptible, and responder), there was no significant difference in the proportion of fish in each category between the different treatments.

**Table 1:** All independent variables for host-parasite metrics analysed via Generalised Linear Models and Linear Mixed Models (GLM and GLMM)

Treatment	Est	Std. error	*p* value
Maximum parasite burden (GLM)			
Bamboo	− 3.15	0.97	**0.001**
Cotton	1.09	1.01	0.28
Polyester	− 1.41	1	0.15
Peak parasite day of (GLM)			
Bamboo	0.17	0.03	** < 0.001**
Cotton	0.02	0.03	0.52
Polyester	0.08	1	**0.01**
Area under curve for (GLM)			
Bamboo	− 0.48	0.26	0.07
Cotton	− 0.42	0.25	0.09
Polyester	− 0.09	0.25	0.71
Rates of parasite increase (GLMM)			
Bamboo	− 1.95	0.85	**0.02**
Cotton	− 1.16	0.81	0.51
Polyester	− 1.28	0.82	0.11

Shown here are the model estimates, standard error, and respective *p* values. Control fish not exposed to any fibres (not shown here) and subsequently infected are the baseline against which all other treatments (i.e. bamboo, cotton, and polyester) are compared within the statistical models. Significant *p* values highlighted in bold

## Discussion

Fibre contamination is now recognised as the major constituent of particulate pollution in marine, freshwater, and terrestrial ecosystems (Horton et al. 2017; Vince and Stoett 2018). In freshwater systems, fibre contaminants are present in multiple fish species (e.g. Silva-Cavalcanti et al. 2017; Su et al. 2019). Despite the prevalence of fibres in freshwater systems, this study is the first to assess the functional impacts of plastic and cellulose-based fibres and associated dye exposure and consumption (polyester, cotton, and bamboo) on fish host-parasite dynamics, specifically host mortality, disease resistance, and off-host parasite survival. For infected fish, whilst polyester and cotton had no major effects on disease dynamics, bamboo was associated with significantly lower maximum parasite burdens. Polyester exposure and/or consumption were also associated with significantly increased mortality of uninfected hosts. Analysis of fibre-based dyes on off-host parasite survival revealed that cotton- and polyester-associated dyes were linked to significantly reduced parasite survival, with the polyester dyes associated with the highest parasite mortalities.

Polyester, specifically polyethylene terephthalate, is the most common thermoplastic polymer used for clothing (Ji 2013) and unsurprisingly most prevalent in multiple surveys of fibre types in fish gastrointestinal tracts (Su et al. 2019). The only studies we are aware off that have tested polyester health effects in fish revealed no significant detrimental effects (Spanjer et al. 2020; Hu et al. 2020). Juvenile Chinook salmon successfully cleared 94% of their gut polyester fibres over 10 days (following in-feed exposure of 20 fibres per food pellet with guts sampled at days 0, 3, 5, 7, and 10) and polyester exposure had no effect on their mean gastrointestinal mass when compared with fish not exposed to any fibres (Spanjer et al. 2020). This Chinook salmon study though was based on short-term exposure and the authors commented that longer term studies may reveal detrimental health effects. In contrast, exposure of adult medaka for 21 days to polyester at levels × 14 greater than those tested in the current study (10,000 fibres/L versus 700 fibres/L) reported denuded epithelium on gill arches, fusion of primary lamellae, and increased mucus production, but no changes in adult growth, weight, or mortality, nor embryonic mortality (Hu et al. 2020). In contrast, the current study which exposed fish to polyester fibres for 52 days demonstrated significantly reduced survival for uninfected hosts, and we acknowledge that longer exposure times may have revealed even more pronounced effects. A plausible reason why polyester was associated with increased mortality of fish and also a limitation of the current study is that polyester fibres were, on average, larger particles than bamboo and cotton fibres. This is certainly linked to the greater physical degradation over time that we observed with cotton and bamboo compared with polyester. Therefore, the larger polyester fibres may have been linked to increased gut blockages and/or morbidities associated with the intestinal lining (e.g. necrosis and lesions), certainly something noted for other microplastic types within fish in previous studies (e.g. polystyrene in Ahrendt et al. 2020). However, in the current study we were unable to assess gut morphology or control for the size of the fibres being released from the commercial fabrics.

When assessing the impacts of fibre-associated dyes on parasite mortality, we observed that cotton and polyester dyes were linked to significantly higher off-host parasite mortalities compared with bamboo dyes and controls. Off-host survival for this *G. turnbulli* strain typically ranges from 2 to 31 h (mean 13.7 h at 25 °C; Schelkle et al. 2013), which was reflected in our control worms (i.e. not exposed to any dyes). Ecological parasitology is increasing our understanding of how pollutants, such as the fabric chemical dyes in this study, impact the health of parasites, making parasites potential indicators of environmental health (reviewed by Vidal-Martínez et al. 2010; Sures et al. 2017). Typically, studies on parasite responses to pollutants have tended to focus on their ability to bioaccumulate toxins, especially heavy metals, particularly for endoparasites (e.g. cestodes and acanthocephalans; Palm and Rückert 2009) and fewer studies have assessed survival of free-living stages of parasites such as trematode cercariae (e.g. Hock and Poulin 2012). For this studies parasite species, *G. turnbulli*, has a direct life cycle (i.e. no intermediate free-living stage); whilst we did reveal impacts of the dyes on the parasite’s direct survival (underlying mechanism unknown), we did not attempt to assess the ability of these monogenean worms to bioaccumulate dyes. Of the three fibre dye types, polyester had the highest number of parasite mortalities and whilst we were unable to tackle the mechanism underlying this increased parasite or host mortality, polyester was leaching a specific dye component (as identified via LCMS analysis; see Supplementary Material) more than bamboo and cotton, and this might have been a contributing factor. The chromatographic analysis also revealed that comparable amounts of dye were released from the bamboo and cotton fibres tested, but for some reason the cotton associated dye/s led to higher parasite mortalities. Current industry practices mean that the chemical identity of fabric dyes tends to remain confidential (Chen and Burns 2006), and unfortunately, we were unable to determine the exact nature of the dye. Whilst it has been shown that wastewater effluent containing textile dyes can be toxic to fish (Zhang et al. 2013; Kaur and Dua 2015), targeting specific fabric dyes and their impacts on fish welfare is much harder without actually knowing the specific dyes in question. However, plausible reasons why fibres and their dyes were associated with increased host and off-host parasite mortality include (1) development of biofilms harbouring infectious agents (Di Pippo et al. 2020; Tang et al. 2020); (2) gastrointestinal-related pathologies (i.e. gut blockage and lesions; Jovanović 2017); (3) toxic effects related to a breakdown product from either the fibres and/or the associated dyes; and/or (4) immune priming associated with microfibres being treated as antigens leading to reduced host survival (Tort 2011).

Unlike the thermoplastic plastic polymer polyester, cotton and bamboo are cellulose-based fibres and with the push to utilise natural alternatives, such as bamboo and hemp (Raj 2021), the biological impacts of these alternatives must be established. Tropical fish, such as guppies used in this study, would naturally consume cellulose-based foliage (Zandona et al. 2011), and the current study revealed that, compared with controls not exposed to any fibres, cotton consumption did not impact disease burden or mortality. Though bamboo is also composed of cellulose, the bamboo utilised in this study was 95% viscose based and therefore technically synthetic in nature (i.e. chemically treated) using regenerated cellulose (structurally the same as natural cellulose; Kauffman 1993) and 5% elastane, which is a synthetic polymer. Elastane is known for its extreme flexibility and hence an essential addition to such clothing, but it is also recognised as non-recyclable and not easily degraded in natura (see Yin et al. 2014). For this study bamboo exposure and consumption was associated with significantly lower maximum parasite burdens compared with fish not exposed to any fibres, whilst also reaching these peaks much later than the control treatments. It is unclear why bamboo consumption was associated with such low maximum parasite burdens, but this could be related to the host immune system being primed to a chronic pollutant leading to a heightened disease response (i.e. increased resistance—see Tort 2011) and/or reduced parasite survival and/or reproduction rate in response to the bamboo fibre and associated dye exposure. However, off-host parasite survival was not affected by direct bamboo dye exposure, leaving potential reproductive changes to the parasite, host immune related effects, or increased host tolerance to the parasites themselves as the likely explanation behind why bamboo may be conferring some degree of protection to the fish.

## Concluding remarks

This study investigated the effects of three fibre types, polyester, cotton, and bamboo and their associated dyes on host-parasite dynamics utilising a freshwater fish model, specifically testing disease resistance, host mortality, and off-host parasite survival. Our results have revealed two key findings. Firstly, polyester fibres were associated with increased host mortality, and polyester and cotton dyes were also linked to increased off-host parasite mortality. Secondly, bamboo fibre exposure and consumption were associated with significantly reduced parasite burdens. With fibre pollution being a dominant form of anthropogenic waste within freshwater environments, studies such as the current one provide important biological assays on potential detrimental impacts on organism welfare.

### Supplementary Information

Below is the link to the electronic supplementary material.

Supplementary file1 (DOCX 402 KB) This paper contains supplementary information in relation to details of statistical analysis outputs relating to survival analysis of host-parasite interactions. Also detailed are the methodology and results of the chromatographic analysis of fibre-based dyes.

## Data Availability

All data pertaining for this manuscript will be made available via the data repository Dryad upon acceptance for publication.
