# Supplementary material for: The impacts of synthetic and cellulose-based fibres and their associated dyes on fish hosts and parasite health
Source: Environ Sci Pollut Res Int. 2023 Nov 13;30(58):121558–68. doi: 10.1007/s11356-023-30794-0 (PMC10724321; doi:10.1007/s11356-023-30794-0)
Supplement: Supplementary file 1 — Supplementary file1 (DOCX 402 KB) This paper contains supplementary information in relation to details of statistical analysis outputs relating to survival analysis of host-parasite interactions. Also detailed are the methodology and results of the chromatographic analysis of fibre-based dyes. [file 11356_2023_30794_MOESM1_ESM.docx]

**Supplementary Material**

The impacts of synthetic and cellulose based fibres and their associated dyes on fish hosts and parasite health

Scott MacAulay, Numair Masud, Josh Davies-Jones, Benjamin D. Ward, Jo Cable

Shown below are statistical analysis output to support the main manuscript analysing the impact of fibre exposure on host-parasite dynamics. Also shown are the details of the chromatographic analysis of fibre-based dyes.

**Host and parasite survival:**

**Survival analysis on the impact of fibre exposure on fish host mortality.**

1. Infected fish Cox’s proportional hazards model: 1) Bamboo coef=-0.302, z=-0.94, p=0.34; 2) Cotton coef=0.28, z=0.88, p=0.37; 3) polyester coef=-0.003, z=-0.01, p=0.99
2. Uninfected fish Cox’s proportional hazards model: 1) Bamboo coef=0.13, z=0.208, p=0.83; 2) Cotton coef=1.33, z=1.87, p=0.06; 3) Polyester coef=2.06, z=2.80, p=0.004

Impact of dye and fibre exposure on parasite (*Gyrodactylus turnbulli*) survival using a parametric survival model: 1) Bamboo std. err=0.12, z=-0.96, p=0.33; 2) Cotton std. err=0.11, z=-2.58, p=0.01; 3) Polyester std. err=0.12, z= -3.15, p=0.001

**Chromatographic analysis of fibre based-chemical dyes**

**Methodology**

To analyse dyes associated with the fibres, chromatographic analysis was conducted on a 1 mL aliquot of the dye-containing water (fibres had been soaked in water for 52 days prior). This water from each sample was added to a clean 2 mL vial, to which we added 100 μL of a 10 mg/mL solution of caffeine (Sigma-Aldrich) in deionised water, as an internal standard (i.e., 1 mg of caffeine per sample). Three dye samples from each fibre type were analysed in a Waters Synapt G2-Si Liquid Chromatography Mass Spectrometry (LCMS) instrument (Everlight Chemical). The triplicate measurements gave consistent readings within a 5% variance, from which we infer that the detection is sufficiently accurate to quantify and characterise the chemical components. The chromatography measurements were performed at 40 °C in a water/acetonitrile (HPLC grade, Fisher Scientific) gradient starting with 99:1 water:acetonitrile with a linear gradient to 2:98 water:acetonitrile over the run time of the measurement. This LCMS technique provided chemical separation of liquid mixtures in tandem with structural identification of individual components. Since the structure and identity of the dyes was not available from commercial sources, due to commercial sensitivity, it was not possible to fully calibrate the LCMS analyses to quantify the dye in the solution. Therefore, the substrate and caffeine peaks were integrated and normalised, i.e., the crude integration data were reported relative to those of the caffeine internal standard peak; the caffeine concentration was constant in each sample under the experimental conditions and the normalisation then ensures that the only variation in signal intensity between samples is caused by a different analyte concentration. This then provides a semi-quantitative measure of the amount of dye leaching into the water.

**Results**

The LCMS revealed that all dyes from the three fibre treatments eluted at 0.66 min, compared to 7.95 min for the internal caffeine control (Figure S1). This short retention time (i.e., 0.66 min) is consistent with a polar species. The normalised integrations were measured as 0.0502, 0.0504 and 0.0638 (each averaged over two replicates) for bamboo, cotton, and polyester respectively (Figure S1 A, B, C), indicating that the bamboo and cotton contain a comparable amount of the colourless dye, whereas the polyester contains approximately 27% more.


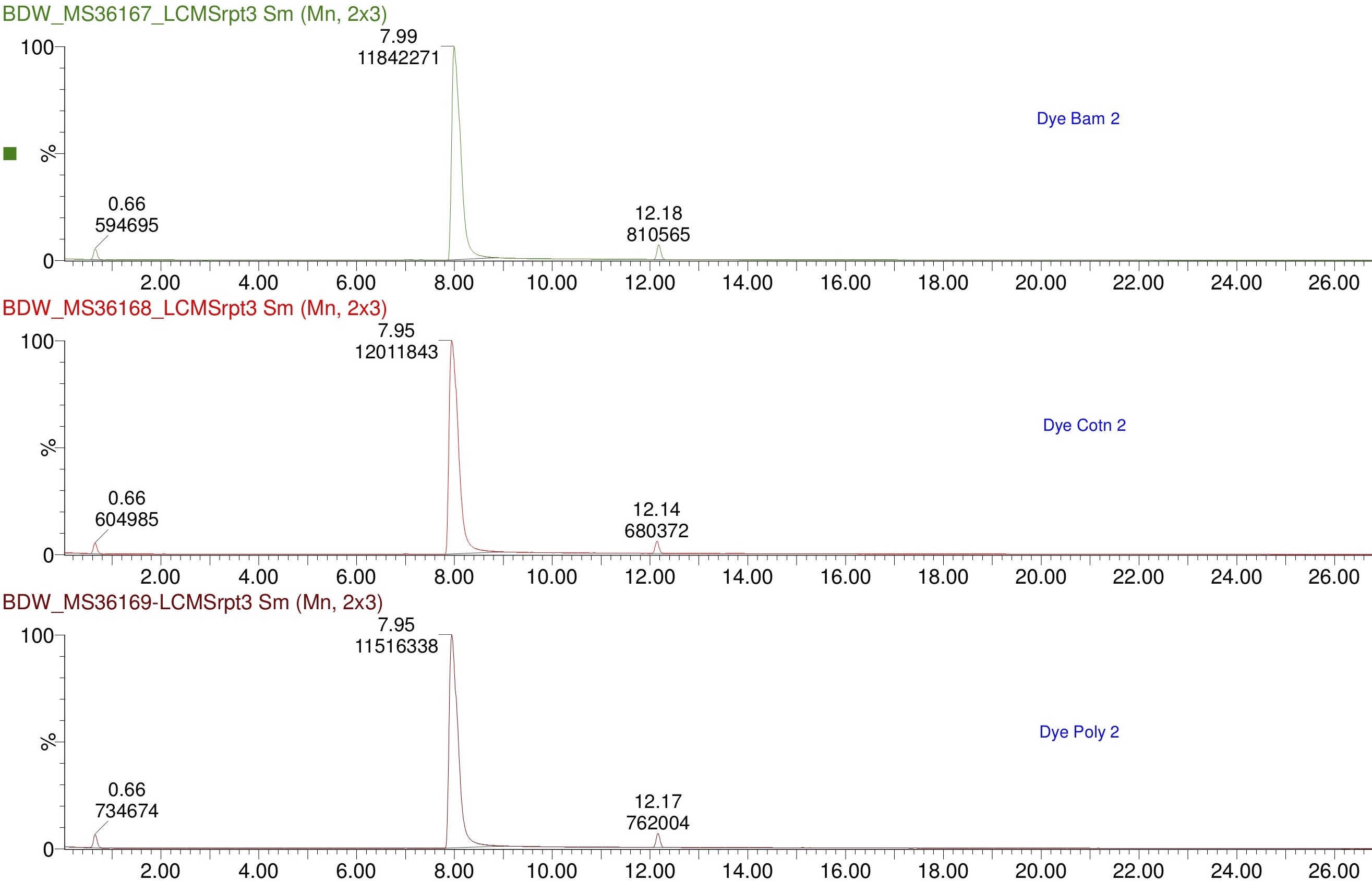


Normalised Signal intensity (%, relative to the highest peak)

Time to elution (minutes)

C: Polyester

B: Cotton

A: Bamboo

**Figure S1.** Chromatograms for A) bamboo, B) cotton, and C) polyester fibres analysed in a Waters Synapt G2-Si Liquid Chromatography Mass Spectrometry (LCMS) instrument (Everlight Chemical). The peaks at 7.95-7.99 and 12.14-12.18 min correspond to the caffeine internal standard.
